# Dyspnea and Wheezing after Adenosine Injection in a Patient with Eosinophilic Bronchitis

**DOI:** 10.1155/2009/356462

**Published:** 2009-11-08

**Authors:** Rodrigo Cartin-Ceba, Marie Christine Aubry, Kaiser Lim

**Affiliations:** ^1^Division of Pulmonary and Critical Care Medicine, Department of Internal Medicine, Mayo Clinic College of Medicine, 200 First Street SW, Rochester, MN 55905, USA; ^2^Division of Anatomic Pathology, Department of Laboratory Medicine and Pathology, Mayo Clinic College of Medicine, 200 First Street SW, Rochester, MN 55905, USA

## Abstract

A 58-year-old nonsmoker female was referred for evaluation of chronic cough of 13 months duration. After an initial work-up, the patient was diagnosed to have chronic cough due to eosinophilic bronchitis. The diagnostic work-up for eosinophilic bronchitis and bronchial biopsy is discussed. Eosinophilic bronchitis is differentiated from asthma. In addition, the patient developed dyspnea, flushing, and wheezing after the administration of adenosine during a cardiac stress test in spite of a negative methacholine challenge. This indirect stimulus of airway hyperresponsiveness suggests the possible involvement of mast cells in eosinophilic bronchitis.

## 1. Introduction

Eosinophilic bronchitis is an uncommon cause of chronic cough in the general practice; however, this entity has been found to be present in 10–20% of patients referred for an investigation by a specialist [1], and recent studies suggest that it can be present in up to 33% of nonsmokers patients referred for chronic cough evaluation [2]. Eosinophilic bronchitis was originally described by Gibson et al. in 1989 [3] and has subsequently been recognized as an important cause of chronic cough. We present the case of a 58-year-old female with chronic cough due to eosinophilic bronchitis complicated by dyspnea, flushing, and wheezing after administration of adenosine for a sestamibi cardiac stress test.

## 2. Case Report

A 58-year-old post menopausal nonsmoker Caucasian female was evaluated for chronic cough of 13 months duration. The patient has a past medical history significant for hyperlipidemia, and obstructive sleep apnea. The cough was described as a dry cough and was severe enough to cause her to gag and vomit. She reported frequent nighttime awakenings due to cough. Initial work-up at another facility was reported as normal pulmonary function tests, negative methacholine challenge test, normal chest radiogram, normal chest and sinus CT scans, and a normal inspection of vocal cords, trachea, and bronchi by flexible bronchoscopy. A bronchial biopsy was performed during the bronchoscopy and results are reviewed below. She was prescribed an empiric one-week trial of prednisone which resulted in near resolution of her cough. The patient was then started on inhaled fluticasone and tiotropium without a clear diagnosis given. As a consequence, she was uncertain about the use of the inhalers and was noncompliant. The cough came back prompting another evaluation. The cough was not associated with rhinorrhea, sneezing, wheezing, dyspnea, postnasal drip, heartburn, chest pain, fever, sputum production, hemoptysis, weight loss, or night sweats. She denied ever having had exposure to immigrants or any travel outside her home state. No history of ACE inhibitors intake was noted. The patient worked with Christmas trees helping to shear, bale, and make wreaths. She has a dog at home but no other pets. She has no prior history of allergies or allergy testing. The patient did not have a history of childhood asthma, sinusitis, GERD, hayfever or tuberculosis, and no history of indoor hot tub. In addition, the patient complained of bilateral sharp chest discomfort for about 10 months, associated with the cough episodes, nonradiating and not associated with exercise, nausea, or diaphoresis.

**Figure 1:**
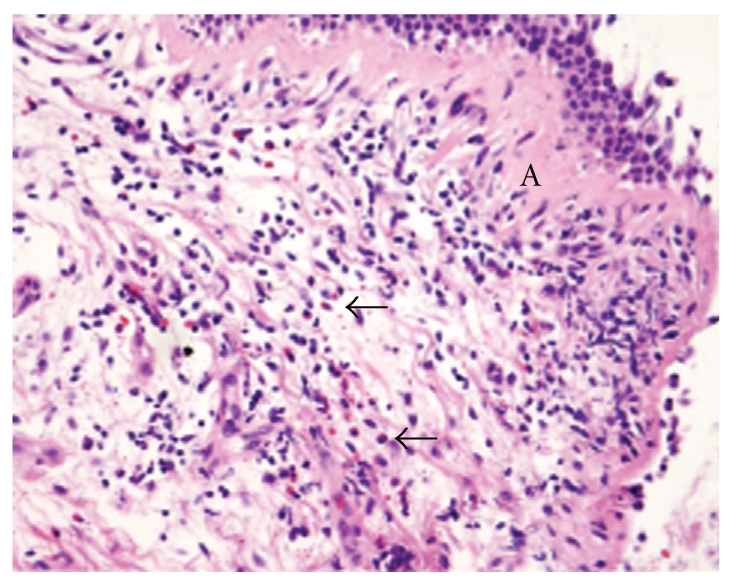
High power photomicrograph of bronchial wall showing mucosa with thickened basal membrane (A) and numerous submucosal eosinophils (arrow), including degranulated forms (Hematoxylin and Eosin; 400X).

Physical examination showed normal vital signs. There was a perforated right tympanic membrane. Oropharynx showed no exudates or lesions, and normal nasal mucosa with no polyps. Lung auscultation showed normal breath sounds, and no wheezing or crackles. The heart rhythm was regular and auscultation evidenced no murmurs, rub, or gallop; her abdomen was soft with no organomegaly, extremities with no peripheral edema, and the skin showed no cyanosis or rash. Finally, no clubbing was observed. Diagnostic work up included a spirometry with FEV1 112% of predicted, FVC 111% of predicted, and an FEV1/FVC ratio of 81. The shape of the inspiratory and expiratory flow-volume curves was unremarkable. The diffusing capacity showed a DLCO of 97% of predicted. The methacholine challenge test showed that the PC20 was >16 mg/dL (normal bronchial responsiveness). Chest CT showed no infiltrates or pleural effusions, and no abnormal hilar or mediastinal lymphadenopathy. CT scan of the sinuses showed normal mucosal thickening and no air-fluid levels. A 24-hour esophageal pH probe of proton pump inhibitor excluded gastroesophageal reflux disease. CBC showed hemoglobin of 13.6 g/dL (normal range 12–15.5 g/dL), the WBC was 9.2 × 10^9^/L (normal range 3.5–10.5 × 10^9^/L), and differential evidenced 60% Neutrophils, 1% eosinophils, 35% lymphocytes, and 4% monocytes. Electrolytes including sodium, potassium, chloride, calcium, magnesium, and phosphorus were within normal limits. Serum creatinine and BUN were 0.8 mg/dL (normal range 0.6–1.1 mg/dL), and 12 mg/dL (normal range 6–21 mg/dL) respectively. Serology for Bordetella pertussis and Respiratory Syncytial virus (RSV) were negative. Her oral exhaled nitric oxide was elevated to 158 parts/billion (upper limit of normal <30 parts/billion). The biopsy of the left main bronchus was reviewed and revealed peribronchiolar chronic inflammation with eosinophils and thickened basement membrane (Figure 1). Sputum examination for eosinophil was not performed. Based on the normal spirometry, negative methacholine challenge test, the elevated oral exhaled nitric oxide that correlates with eosinophilic airway inflammation [4, 5] and bronchial biopsy, a diagnosis of chronic cough due to eosinophilic bronchitis was made. To exclude the possibility of a cardiac cause of her chest discomfort, the patient underwent an adenosine sestamibi study. During this test, she developed sudden onset of dyspnea, flushing, and bilateral wheezing (confirmed by two different clinicians) during the adenosine infusion and required hospitalization in the intensive care unit, where she was successfully treated with intravenous aminophylline.

## 3. Discussion

The diagnosis of eosinophilic bronchitis is usually made in patients with chronic cough and airway eosinophilia in the absence of dyspnea, wheezing, airflow limitation, and lack of airway hyperresponsiveness measured by the methacholine challenge test. When facing a patient with chronic cough, it is always very important to exclude other common causes of chronic cough such as gastroesophageal reflux disease, asthma, or upper airway cough syndrome. Asthma, cough variant asthma, and EB share many features including chronic cough and airway eosinophilia (≥3% eosinophils in the sputum, increased eosinophils in bronchial biopsy, or oral exhaled nitric oxide >30 parts/billion). However, the hyperresponsiveness provocative test with methacholine is positive with asthma or cough variant asthma; whereas it is negative with EB and helps to differentiate these entities (Table 1).

Erlich described the eosinophil more than 100 years ago and its presence in the sputum was initially recognized as an important marker in asthma [6]. Eosinophils are closely associated with immune responses due to the activation of Th2 lymphocytes through the production of specific eosinophilic growth factors such as IL-5, IL-4, and IL-13 [7]. As stated above, the main difference of EB and asthma lies in the lack of airway hyperresponsiveness seen in EB with direct stimuli to measure bronchial hyperresponsiveness such as methacholine challenge test.

The histological changes described in the biopsy of EB are seen also in asthma and cough variant asthma, and a similar Th2 cytokine driven airway inflammation has been proposed in both entities [8, 9]; however, as stated above, airway hyperresponsiveness and variable airflow obstruction, which are the defining features of asthma, are not present in eosinophilic bronchitis. Similar levels of IL-4 and IL-5 have been found in both asthma and eosinophilic bronchitis [9], but recent studies showed that levels of IL-13 are significantly higher in asthmatic patients than patients with eosinophilic bronchitis, who have levels of IL-13 similar to healthy subjects [7].

The presence of elevated oral exhaled nitric oxide (eNO) has been correlated with predominantly eosinophilic airway inflammation and can be seen in both asthma and EB [4, 5]. Treatment with inhaled corticosteroids usually reduces the level of eNO in both entities [5]. Eosinophilic bronchitis responds well to treatment with inhaled corticosteroids in the short term, with a reduction in symptoms and fall in sputum eosinophil count [10]; however, the long-term outcome of the disease is unclear. In a 10-year followup study of the eight patients originally described by Gibson, complete resolution of symptoms was the most common outcome but a minority of patients had developed fixed airflow obstruction [11]. Brightling et al. have also described a patient with eosinophilic bronchitis who developed fixed airflow obstruction in association with prolonged uncontrolled eosinophilic airway inflammation [1]. Others have speculated that eosinophilic bronchitis is an early stage in the development of an asthma phenotype [12] although there is less evidence that evolution to more typical asthma occurs. A more recent study of 32 patients, who were initially diagnosed as eosinophilic bronchitis and were followed for more than 1 year with inhaled corticosteroid therapy, showed that 3 patients developed symptoms consistent with asthma and had a positive methacholine challenge test after an initial negative one, 5 patients developed fixed airflow obstruction and one patient remained symptom free after stopping the therapy; the remaining patients presented ongoing milder symptoms [13].

Our patient developed symptoms and signs suggestive of airway hyperresponsiveness after the administration of intravenous adenosine for the cardiac stress test that was ordered to evaluate her atypical chest pain. Adenosine type 2 receptors are found in the mast cells and when stimulated can potentiate the histamine release from these cells and therefore precipitate bronchoconstriction [14]. Interestingly, histamine and prostaglandin D2 concentrations have been found to be increased in EB when compared with asthma, suggesting that the activation of mast cells in the superficial airway mucosa may differ in these two diseases [15]. Patients with eosinophilic bronchitis show variably bronchial hyperresponsiveness to nebulized adenosine with 6 out of 9 being negative [16]. Response to indirect stimuli of airway hyperresponsiveness has not been well described in EB. Patients with EB do not manifest airway hyperresponsiveness with direct stimuli that is, methacholine. Adenosine stress test for cardiac ischemia should be reconsidered in patients with chronic cough if eosinophilic bronchitis is suspected based on our report. This is the first description of symptoms and signs suggestive of airway hyperresponsiveness associated with adenosine injection in a patient with known eosinophilic bronchitis.

## 4. Conclusion

Eosinophilic bronchitis is a common cause of chronic cough in referral centers. It shares similarities with asthma such as increased eosinophils in the airways, increased IL-4 and IL-5, and improvement of symptoms with inhaled corticosteroids; however, eosinophilic bronchitis differs from asthma because it presents with normal pulmonary function and normal airway responsiveness. We described the first case of airway hyperresponsiveness after systemic administration of adenosine in a patient with eosinophilic bronchitis presenting as chronic cough.

## Figures and Tables

**Table 1:** Comparison of clinical findings and tests between asthma, cough variant asthma, and eosinophilic bronchitis.

	Wheezing	Cough	Bronchoprovocation testing (airway hyperresponsiveness)	Eosinophils in sputum	Exhaled oral nitric oxide	Response to inhaled steroids
Asthma	Common symptom	May be present	Positive methacholine challenge test	Sputum eosinophilia (≥3%)	>30 parts/billion	Improved symptoms
Cough variant asthma	Absent	Always present	Positive methacholine challenge test	Sputum eosinophilia (≥3%)	>30 parts/billion	Improved symptoms
Eosinophilic bronchitis	Absent	Always present	Negative methacholine challenge test	Sputum eosinophilia (≥3%)	>30 parts/billion	Improved symptoms
